# High temperatures and low humidity promote the occurrence of microsporidians (Microsporidia) in mosquitoes (Culicidae)

**DOI:** 10.1186/s13071-024-06254-0

**Published:** 2024-04-11

**Authors:** Artur Trzebny, Olena Nahimova, Miroslawa Dabert

**Affiliations:** 1 Molecular Biology Techniques Laboratory, Faculty of Biology, Adam Mickiewicz University, Poznan, Poland; 2 Genetics and Cytology Department, School of Biology, V.N. Karazin Kharkiv National University, Kharkiv, Ukraine

**Keywords:** Metabarcoding, Next-generation sequencing, Mosquito vectors, Parasitic infections, Global warming, Environmental indicators

## Abstract

**Background:**

In the context of climate change, a growing concern is that vector-pathogen or host-parasite interactions may be correlated with climatic factors, especially increasing temperatures. In the present study, we used a mosquito-microsporidian model to determine the impact of environmental factors such as temperature, humidity, wind and rainfall on the occurrence rates of opportunistic obligate microparasites (Microsporidia) in hosts from a family that includes important disease vectors (Culicidae).

**Methods:**

In our study, 3000 adult mosquitoes collected from the field over 3 years were analysed. Mosquitoes and microsporidia were identified using PCR and sequencing of the hypervariable V5 region of the small subunit ribosomal RNA gene and a shortened fragment of the cytochrome *c* oxidase subunit I gene, respectively.

**Results:**

DNA metabarcoding was used to identify nine mosquito species, all of which were hosts of 12 microsporidian species. The prevalence of microsporidian DNA across all mosquito samples was 34.6%. Microsporidian prevalence in mosquitoes was more frequent during warm months (> 19 °C; humidity < 65%), as was the co-occurrence of two or three microsporidian species in a single host individual. During warm months, microsporidian occurrence was noted 1.6-fold more often than during the cold periods. Among the microsporidians found in the mosquitoes, five (representing the genera *Enterocytospora*, *Vairimorpha* and *Microsporidium*) were positively correlated with an increase in temperature, whereas one (*Hazardia* sp.) was significantly correlated with a decrease in temperature. Threefold more microsporidian co-occurrences were recorded in the warm months than in the cold months.

**Conclusions:**

These results suggest that the susceptibility of mosquitoes to parasite occurrence is primarily determined by environmental conditions, such as, for example, temperatures > 19 °C and humidity not exceeding 62%. Collectively, our data provide a better understanding of the effects of the environment on microsporidian-mosquito interactions.

**Graphical Abstract:**

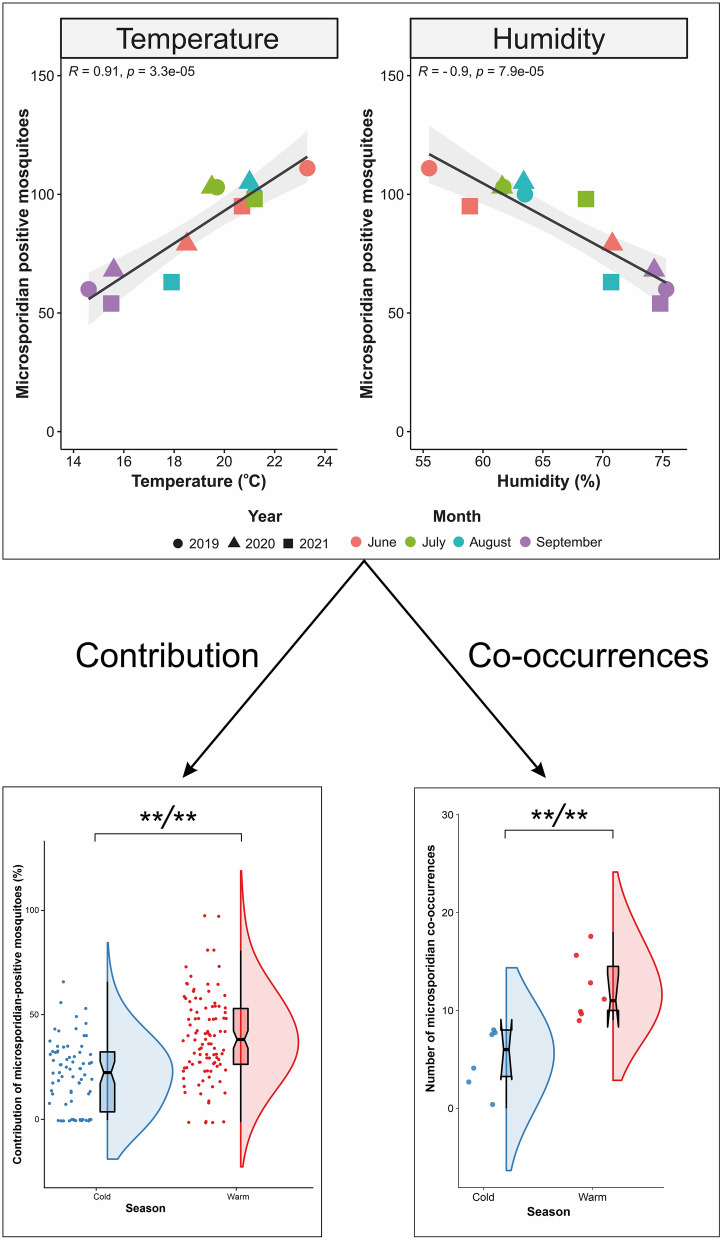

**Supplementary Information:**

The online version contains supplementary material available at 10.1186/s13071-024-06254-0.

## Background

Mosquitoes (Culicidae) are among the most important disease vectors worldwide. Although the exact number of mosquito species relevant to human health is unknown, a recent extensive literature survey indicated that approximately 2.5% of the 3578 mosquito species are known vectors for 78 human disease-causing agents and that an additional 243 species (6.8%) were identified as potential or likely vectors [1]. However, mosquitoes are vulnerable to parasitic infections. Microsporidians (Microsporidia) are among the most common mosquito parasites, and can cause mortality in mosquito larvae and adults [2].

Microsporidia are obligate intracellular eukaryotic parasites distributed worldwide that can infect nearly all animal phyla [3]. Among the 1700 described microsporidian species [4], over 250 belonging to 34 genera have been reported in mosquitoes [5]. Microsporidian spores, the only developmental stage with the ability to survive outside the host cell, can be transmitted horizontally, when released in faeces or when an infected host dies [6–8], or vertically, by infecting the ovaries and associated reproductive structures of the host [9, 10].

Microsporidian parasitism has several effects on individual mosquitoes. Infection during the larval phase results in impaired larval development and the subsequent failure to develop into adult mosquitoes. Infection leads to a reduction in the body size of the host and in the levels of lipids, glycogen and sugars. In addition, infected females are more likely to die and significantly less likely to feed. Moreover, these parasites may manipulate both the composition of the microbiome and the immune response of mosquitoes [11–15].

However, reports on the effects of microsporidians on mosquito-vectored pathogens are limited. More effective melanisation and less successful infection by *Plasmodium* (Apicomplexa) observed in microsporidian-infected mosquitoes compared to uninfected ones suggests that microsporidians impede the development of apicomplexans by priming the immune system of the mosquito [16]. A recent study demonstrated that infection by a microsporidian, designated *Microsporidia* MB, impairs *Plasmodium falciparum* transmission in *Anopheles arabiensis* [14] by reducing the establishment of *Plasmodium* oocysts in the *Anopheles* midgut and impeding the colonisation of mosquito salivary glands by *Plasmodium* sporozoites. The widespread distribution of *Microsporidia* MB among *Anopheles* mosquitoes makes this microsporidian an appealing candidate for controlling parasite transmission in West Africa [17].

Several factors influence microsporidian infection and host cell development [18]. One basic mode of infection is to penetrate and remain in the host intestine long enough to germinate [19]. Additionally, specific protein–protein interactions between polar tube proteins or spore wall proteins and host cell receptors are required for microsporidians to invade host intestinal cells [20–22]. In addition, the host strain [23–28], developmental stage [27, 29–31] and sex [32–34] affect microsporidian infections and determine the level of host resistance to these pathogens. Moreover, it has been shown that temperature can affect microsporidia growth by influencing the number of spores produced as well as increasing microsporidia infection [35–41].

Owing to climate change, concerns are growing that mosquito-pathogen interactions may correlate with climatic factors [42, 43]. The importance of temperature in understanding mosquito population dynamics has been addressed in studies concerning *Plasmodium* risk in malaria mosquitoes [44–48]. Indeed, climatic parameters such as temperature, humidity and rainfall significantly influence both mosquito life history traits and pathogen development within their bodies [49–54]. For example, it has been demonstrated that temperature has a significant effect on mosquito host-seeking behaviour, development, geographic range, survival and competence to transmit pathogens [49, 50, 53–57]. There is also evidence that humidity affects mosquito dispersal, longevity, egg-laying and feeding behaviour [58, 59]. Rainfall can alter the availability of suitable larval habitats, affecting egg and larval viability [60, 61]. Finally, wind also affects mosquito dispersal and migration [62, 63].

Little is known regarding the influence of climatic factors on the prevalence and development of microsporidians in hosts. The results of a recent study and, to the best of our knowledge, the only study involving mosquitoes, suggest that a gradual decrease in ambient temperature postpones the growth and development of *Parathelohania iranica* (Microsporidia: Amblyosporidae) in the affected anopheline larvae [64]. Therefore, the aim of the present study was to analyse the impact of environmental factors such as temperature, humidity, wind and rainfall on the microsporidian occurrence rates of these important disease vectors.

## Methods

### Mosquito sample and meteorological data

The mosquito samples analysed in this study consisted of 3000 adult individuals, including 1500 females and 1500 males, collected from June to October 2019, 2020 and 2021 from the periphery of a mixed hornbeam-oak forest surrounding Rusalka Lake, located in the northwestern part of the city of Poznan, western Poland (N 52.426389, E 16.877778). Mosquitoes were collected using a U.S. Centres for Disease Control and Prevention light trap (CDC-LT; Centres for Disease Control and Prevention, Atlanta, GA, USA) and a human landing catch, and were preserved in 80% ethanol at 4 °C until DNA extraction.

Meteorological data were provided by the Institute of Meteorology and Water Management, National Research Institute, Warszawa, Poland (available at https://dane.imgw.pl). Measurements were performed 2 m above the ground. Average monthly temperature, humidity, wind speed and rainfall were calculated based on daily measurements (Additional file 1: Tables S1, S2). Notably, during the study period, the humidity decreased as the temperature increased (*R* = − 0.57; *p* < 0.001). No other significant relationships were found between the environmental variables. (Additional file 1: Table S1; Additional file 1: Fig. S1).

### DNA extraction

To detect potential contamination of mosquito surfaces with microsporidians, mosquitoes were washed with 96% ethyl alcohol that served as a washing extraction. The washing solution was then subjected to DNA extraction. The washing solution was first filtered through the 0.22-µm pore MF-Millipore Membrane Filter (Merck KgaA, Darmstadt, Germany), following which the filter was cut and placed in 180 μl of ATL lysis buffer (Qiagen, Hilden, Germany) and incubated with 0.2 mg of Proteinase K (Bio Basic Inc., Markham, ON, Canada) for 48 h at 56 °C. Next, 100 μl of the lysate was used for DNA extraction using the DNeasy Blood & Tissue Kit (Qiagen) according to the manufacturer’s protocol for animal tissues.

Mosquito total genomic DNA was extracted using a modified ammonium hydroxide method [65]. Each mosquito was separately homogenised in 200 μl of 0.7 M ammonium hydroxide (POCH S.A., Gliwice, Poland) for 30 s using a Pellet Cordless Motor instrument (DWK Life Sciences, Wertheim, Germany) with disposable micropestles (Scientific Specialties Inc., Lodi, CA, USA). Samples were incubated for 20 min at 99 °C with shaking, and then the tubes were opened and further left under the same conditions for approximately 5 min to concentrate the lysate to approximately 100 μl. The samples were then centrifuged for 5 min at 10,000 rpm, and the supernatant was collected. Prior to PCR analysis, the DNA extracts were normalised with sterile water to a concentration of approximately 10 ng/µl. Negative controls from blank DNA extractions and PCR reagents were included in each PCR and analysed in the same manner as the mosquito sample.

The mini-COI marker, covering approximately 370 bp from the 5ʹ end of the cytochrome *c* oxidase subunit I (COX1) gene, was amplified using the primer pair bcdF01 (CATTTTCHACTAAYCATAARGATATTGG) [66] and bcdR06 (GGDGGRTAHACAGTYCAHCCNGT) [67] tailed at the 5ʹ ends with double indexed adapters (forward tail CCATCTCATCCCTGCGTGTCTCCGACTCAG-index-GAT; reverse tail CCTCTCTATGGGCAGTCGGTGAT-index) for sequencing using the Ion Torrent system (Life Technologies, Thermo Fisher Scientific, Waltham, MA, USA). PCR amplification was performed in a reaction volume of 5 µl containing Hot FIREPol DNA Polymerase (Solis BioDyne, Tartu, Estonia), each tailed primer at 0.25 µM and 1 µl of template DNA. The amplification program was set as follows: 12 min at 95 °C, followed by 35 cycles of 15 s at 95 °C, 30 s at 50 °C and 45 s at 72 °C, with a final extension step at 72 °C for 5 min.

The hypervariable V5 region, covering approximately 200 bp of the small subunit ribosomal rRNA gene (SSU rDNA), was amplified in two technical replicates using the microsporidian-specific primer sets CM-V5F (GATTAGANACCNNNGTAGTTC) and CM-V5R (TAANCAGCACAMTCCACTC) [67]. The PCRs were performed in a total volume of 10 µl containing Hot FIREPol DNA Polymerase (Solis BioDyne), each tailed primer at 0.25 µM, and 1 µL of template DNA. The amplification program was set as follows: 12 min at 95 °C, followed by 35 cycles of 15 s at 95 °C, 30 s at 50 °C and 30 s at 72 °C, with a final extension step at 72 °C for 5 min.

### Library construction and NGS sequencing

For each PCR, 3 µl of DNA solution was electrophoresed in a 2% agarose gel to check amplification efficiency. SSU rDNA and mini-COI libraries were prepared separately. Next, the amplicons were pooled and purified using a 2% E-Gel SizeSelect II Agarose Gel System (Invitrogen, Thermo Fisher Scientific) according to the manufacturer’s protocol. The DNA concentration and fragment length distribution of the libraries were determined using a High-Sensitivity D1000 Screen Tape assay on a 2200 Tape Station system (Agilent Technologies, Inc., Santa Clara, CA, USA). Clonal template amplifications were performed using the Ion Torrent One Touch System II and Ion Torrent OT2 Kit (Life Technologies, Thermo Fisher Scientific) according to the manufacturer’s protocol. For emulsion PCR, SSU rDNA and mini-COI libraries were pooled in a 10:1 ratio. Sequencing was performed using the Ion 540 Kit-OT2 and Ion S5 systems on Ion 540 chips (Life Technologies, Thermo Fisher Scientific), according to the manufacturer’s instructions. Sequencing was designed to yield approximately 10,000 and 1000 reads per SSU and COI amplicons, respectively.

### Read processing and data analysis

Raw sequencing data were prefiltered using the Ion Torrent Suite software version 5.18.1 (Life Technologies, Thermo Fisher Scientific) to remove polyclonal and low-quality sequences. Further bioinformatics analysis was conducted using the fastq data. Sequence reads shorter than 180 bp were removed from the dataset using Geneious Prime 2023.1.2 (Biomatters, Inc., Boston, MA, USA). The FastX-Toolkit [68] was used to extract sequences with a minimum of 50% of bases having a quality score ≥ 25. Quality-filtered sequences were separated into individual combinations of indices using the Geneious Prime software. Next, the sequences were trimmed at the 5ʹ and 3ʹ ends to exclude the PCR primers. Sequences were denoised to generate amplicon sequencing variants (ASVs) using the DADA2 denoise-pyro method implemented in QIIME2 version 2023.5 [69, 70]. The UNCROSS2 algorithm was used to remove ASVs detected in control samples from the dataset [71]. ASVs were compared to those in GenBank using the Basic Local Alignment Search Tool for Nucleotides (BLASTN) [72] (access date: September 2023), optimised for highly similar sequences (MegaBlast algorithm) [73]. ASVs were compared to GenBank using a 97% identity threshold to determine mosquito species and 100% identity to identify microsporidian species, as described in previous studies [5, 67, 74].

### Phylogenetic analyses

To confirm the taxonomic affiliation of the 13 microsporidian rDNA sequences detected in this study, an additional 78 SSU rDNA sequences representing all known microsporidian lineages were used for phylogenetic analysis [5, 75]. Sequences were aligned using the L-INS-i algorithm in MAFFT v. 7.450 [76, 77] as implemented using Geneious Pro software (Biomatters, Inc.). The final alignment consisted of 2888 nucleotide positions. The best-fit model of DNA evolution (GTR + I + G) was selected using PartitionFinder2 software [78]. Phylogenetic trees were constructed using maximum likelihood (ML) in Garli version 2.0 [79] and Bayesian inference (BI) in MrBayes 3.2.6 [80]. Each BI run of the four independent chains was performed in 2 × 20,000,000 generations and trees were sampled every 1000 generations. The final consensus tree was generated after discarding a burn-in fraction of 0.25% of the initial trees, and the average standard deviation of the split frequencies dropped below 0.002. Bootstrap support for the ML tree was calculated using 1000 data replicates as implemented using Garli [79]. Trees were edited using FigTree 1.4.4 [81] and CorelDRAW 2021 (Alludo, Ottawa, ON, Canada). Taxonomic names of the microsporidian clades were assigned as previously described [75, 82–84].

### Statistical analyses

Pearson’s correlation coefficient (*r*) [85] was calculated to determine the correlations between environmental factors, such as temperature, humidity, wind and rainfall. Spearman’s correlation coefficient (rho [ρ]) [86] was calculated to determine correlations between both mosquitoes and the same environmental factors. Spearman’s and Pearson’s correlation results were visualised using Tidyverse v. 1.3.0 [87] and ggplot2 v. 3.35 [88] software packages. Comparisons between independent groups were conducted using a two-way analysis of variance (ANOVA), considering warm/cold months and mosquito sex [89, 90]. The ANOVA results were visualised using the ggplot2 v. 3.35 package [88]. Indicator species analysis [91] was performed to determine whether microsporidian species were exclusively found during a specific season and whether these microsporidian species were commonly found in certain treatment groups, as revealed by the A and B components of the indicator species analysis. Indicator species analysis with 9 × 10^10^ permutations was performed using the multipatt function [91, 92] in the indicspecies package version 1.7.9 [91]. McNemar’s Chi-squared test [93] was used to assess the relationship between mosquito sex, occurrence of microsporidians, and microsporidian co-occurrence. An UpSet plot was generated using the UpSetR v. 1.4.0 [94] and ggplot2 v. 3.35 [88] software packages.

## Results

### Seasonal dynamics of mosquitoes

**Fig. 1:**
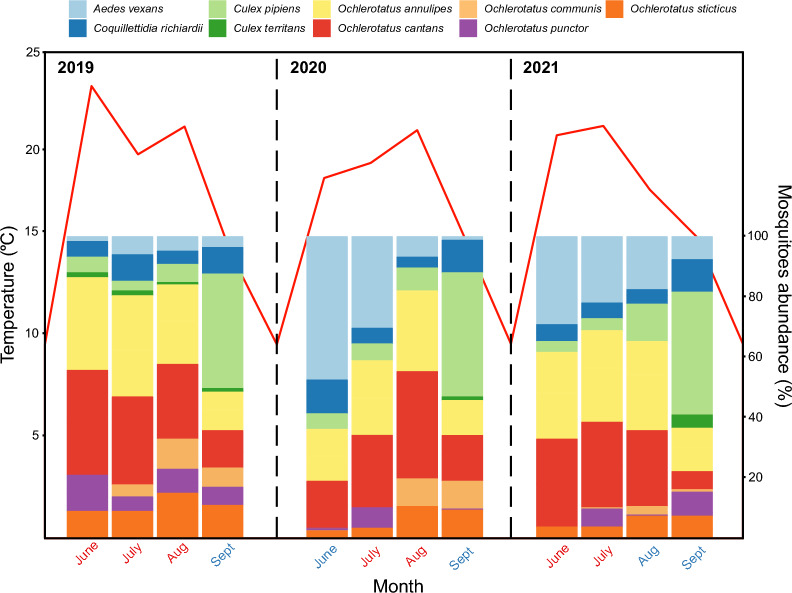
The abundance of each mosquito species in the samples used in this study (right axis). Mosquito species are indicated by the legend at the top of the figure. The red line shows the average monthly temperature (left axis). Months are marked as cold (blue) and warm (red). Cold months were defined as temperature < 19 °C and humidity > 65%; warm months were defined as temperature > 19 °C and humidity < 65%

Using mini-COI data, all mosquitoes were unambiguously assigned to nine species common to Central Europe: *Aedes vexans* (*n* = 446); *Coquillettidia richiardii* (221);* Culex pipiens* (432) and * Cx. territans* (27); and *Ochlerotatus annulipes* (718), *O. cantans* (701), *O. communis* (107), *O. punctor* (159) and *O. sticticus* (199) (Fig. 1; Additional file 1: Table S3). The representative sequences are available in GenBank (Additional file 1: Table S4).

*Ochlerotatus annulipes* and *O. cantans* were most abundant (≥ 24%) in months when the average monthly temperatures were > 19°C and humidity did not exceed 62% (Fig. 1; Additional file 1: Tables S3, S5). In addition, with higher temperature and lower humidity, more individuals of *O. annulipes* (ρ_temp._ = 0.83, *p*_temp_ < 0.001; ρ_humid._ = — 0.76, *p*_humid_ = 0.004) and *O. cantans* (ρ_temp._ = 0.87, *p*_temp_ < 0.001; ρ_humid._ = — 0.84, *p*_humid_ = 0.004) were observed. This relationship was observed in both females and males (Additional file 1: Table S6).

When the temperature decreased to < 16 °C and humidity reached > 70%, the dominant (≥ 38%) species was *Cx. pipiens* (Fig. 1; Additional file 1: Tables S3, S5). Thus, its occurrence was correlated with a decrease in temperature (ρ_temp._ = − 0.89, *p*_temp_ < 0.001) and an increase in humidity (ρ_humid._ = 0.76, *p*_humid_ = 0.005) (Additional file 1: Table S6). Moreover, similar relations were observed among both *C. richiardii* females (ρ_temp._ = − 0.8, *p*_temp_ = 0.001; ρ_humid._ = 0.72, *p*_humid_ = 0.009) and *Cx. territans* males (ρ_temp._ = − 0.83, *p*_temp_ = 0.002; ρ_humid._ = 0.7, *p*_humid_ = 0.01) (Additional file 1: Table S6).

A slightly higher proportion of female and male *A. vexans* (ρ_wind._ = 0.56, *p*_wind_ = 0.04; ρ_wind._ = 0.6, *p*_wind_ = 0.04) and lower proportion of *O. punctor* females (ρ_rain_ = − 0.78, *p*_rain_ = 0.03) during higher rainfall (Additional file 1: Table S6) were observed. Wind did not affect mosquito abundance (Additional file 1: Table S6).

### Seasonal dynamics of microsporidians

Sequencing of the SSU rRNA gene fragment revealed 12 microsporidian species: *Amblyospora salinaria*, *A. stimuli*, *Amblyospora* sp. 1 (identical to the sequences under GenBank acc. no. AY090055), *Amblyospora* sp. 2 (identical to MT118722); *Hazardia* sp. (identical to AY090066); *Encephalitozoon hellem*; *Enterocytospora artemiae*; *Microsporidium* sp. BLAT1 and *Microsporidium* sp. PL01; and three species belonging to the genus *Vairimorpha* (previously classified as *Nosema* [84]): *V*. *adaliae*, *V. ceranae* and *Vairimorpha* sp. CHW−2007a. The microsporidians found in the present study belonged to three of the five major clades: Amblyosporidia (*Hazardia*, *Amblyospora*), Enterocytozoonida (*Enterocytospora*, *Microsporidium* BLAT1, *Microsporidium* PL01) and Nosematida (*Encephalitozoon*, *Vairimorpha*) (Additional file 1: Fig S2; Additional file 1: Table S7).

**Fig. 2:**
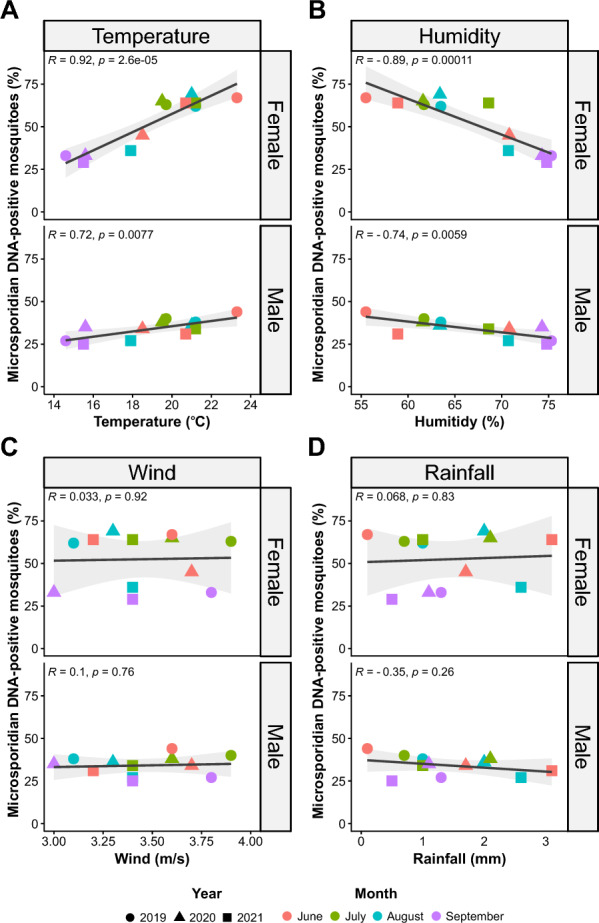
Correlation plots between microsporidian DNA-positive mosquitoes and **a** temperature (°C), **b** humidity (%), **c** wind (m/s) and **d** rainfall (mm). The* R* value indicates the Pearson’s correlation coefficient statistic, the* p* value is statistically significant, the shadowed area shows the 95% confidence interval, and the black line is the regression line. The month and year of the data points are indicated according to the legend at the bottom

In total, 34.6% (1039/3000) of the mosquitoes tested positive for microsporidian DNA, representing all mosquito species detected in this study (Additional file 1: Tables S8, S9). The number of microsporidian-positive mosquitoes significantly correlated with both an increase in temperature (Additional file 1: Fig. S3A) and a decrease in humidity (Additional file 1: Fig. S3B). Overall, for both environmental factors, a linear relationship was observed with a coefficient adjustment of *R* = 0.9 (*p* ≤ 0.001) and *R* = − 0.9 (*p* ≤ 0.001), respectively. This dependence was observed in both females and males; however, it was stronger in females (*R* =  ± 0.9) and slightly weaker in males (*R* =  ± 0.7) (Fig. 2a, b). The remaining environmental factors were not significant, with values of 0.054 (*p* = 0.87) and − 0.047 (*p* = 0.89) for wind and rainfall, respectively (Fig. 2c, d;, Additional file 1: Fig. S3C, S3D).

**Fig. 3:**
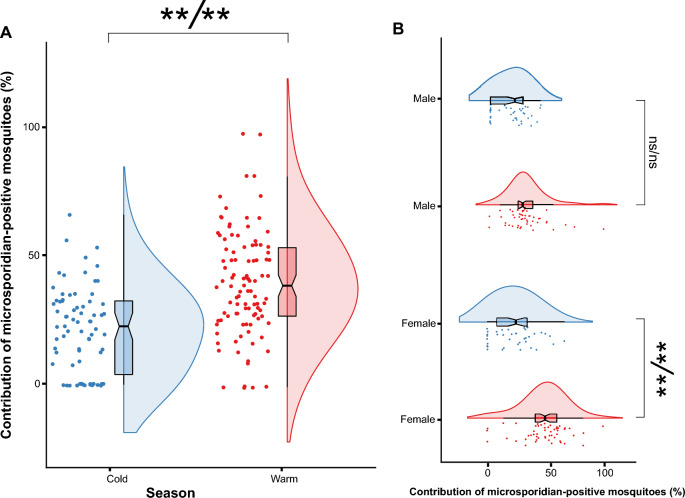
Raincloud plots (i.e. combined violin plots, box plots and dot plots) for the contribution of infected mosquitoes relative to cold or warm months: **a** for the mosquito pool without dividing into females and males and **b** separately for females and males, collected in cold (blue) and warm (red) months. The dot plots show the proportion of infected mosquitoes (jittered horizontally). Each dot is the individual proportion of a particular species with a particular sex in a single month. The box plots show the extremes (whisker tails), interquartile range (box boundaries) and median (horizontal line). The violin plots show the probability density of the data. Symbols (asterisks or 'ns') indicate Bonferroni* p*-value (B) and Holm* p*-value (H) (B/H). Double asterisks (**) indicate statistical significance at *p* < 0.01; ns, no statistical significance

Microsporidian occurrence was noted 1.6-fold more often during the warm months than during the cold periods (Fig. 3a; Additional file 1: Table S8). During the cold months, both females and males were microsporidian positive at similar rates of 28.2% and 23.7%, respectively (Fig. 3b; Additional file 1: Table S9). However, a slightly higher fraction of microsporidian-positive males (29.8%) and more than half of the females (51.9%) were microsporidian positive during warm periods (Additional file 1: Tables S10, S11), and this difference was statistically significant (Fig. 3b; Additional file 1: Tables S10, S11).

Because the presence of microsporidian DNA does not necessarily indicate an infection, we excluded *E. hellem* and *V. ceranae* as infection factors (detected in < 1% of all individuals analysed: 0.1% and 0.57%, respectively), and their ASVs were covered by low numbers of reads (< 50). With the exception of *E. hellem* and *V. ceranae*, each microsporidium species was found in at least five different mosquito species (Additional file 1: Fig. S4).

**Fig. 4:**
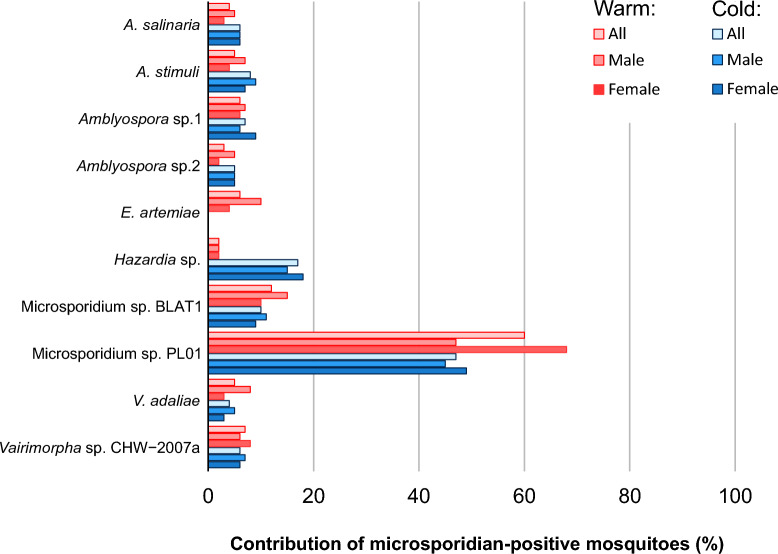
Occurrence of microsporidian species found in total (All), male and female mosquitoes collected during the cold and warm months. Comparative statistics are presented in Additional file 1: Tables S12 and S13

Almost all microsporidian species occurred more frequently during the warm months (Fig. 4). We observed *En. artemiae* in mosquitoes during the warm months; moreover, six microsporidian species, *Amblyospora* sp.1 and *En. artemiae* and *Microsporidium* sp. BLAT1, *Microsporidium* sp. PL01, *V. adaliae,* and *Vairimorpha* sp. CHW−2007a, were significantly more frequent during the warm season (0.001 ≤ *p* ≤ 0.035) (Additional file 1: Table S12). *Hazardia* sp. was the only species that occurred significantly more frequently during the cold months (*p* = 0.001) (Additional file 1: Table S12). Indicator species analysis showed that *En. artemiae* (A component = 1; *p* = 0.005) can be considered to be an indicator of microsporidia in the warm months, whereas *Hazardia* sp. (A component = 0.86; *p* = 0.005) can be considered to be an indicator of microsporidians in the cold months (Additional file 1: Table S13).

During warm months, significant differences in prevalence were found between microsporidian-positive females and males for *Microsporidium* sp. PL01 (*p* < 0.001) and *Vairimorpha* sp. CHW−2007a (*p* < 0.001), with the prevalences being higher in females than in males by approximately 2.8- and 2.4-fold, respectively (Fig. 4; Additional file 1: Table S12). No variations were observed in the abundance of microsporidian-positive females and males for any microsporidian species detected in the mosquitoes during the cold months (Fig. 4; Additional file 1: Table S12).

Additionally, among the identified microsporidians, mixed temperature- and sex-dependent interactions were observed for *Microsporidium* sp. PL01, and *Vairimorpha* sp. CHW−2007a (*F*_A_ = 30.91, *p* < 0.001;* F*_A_ = 6.84, *p* < 0.001). In both cases, the observed effect size η2 was large at the 0.78 and 0.32 levels, respectively (Additional file 1: Table S12).

### Impact of environmental factors on microsporidian occurrence

**Fig. 5:**
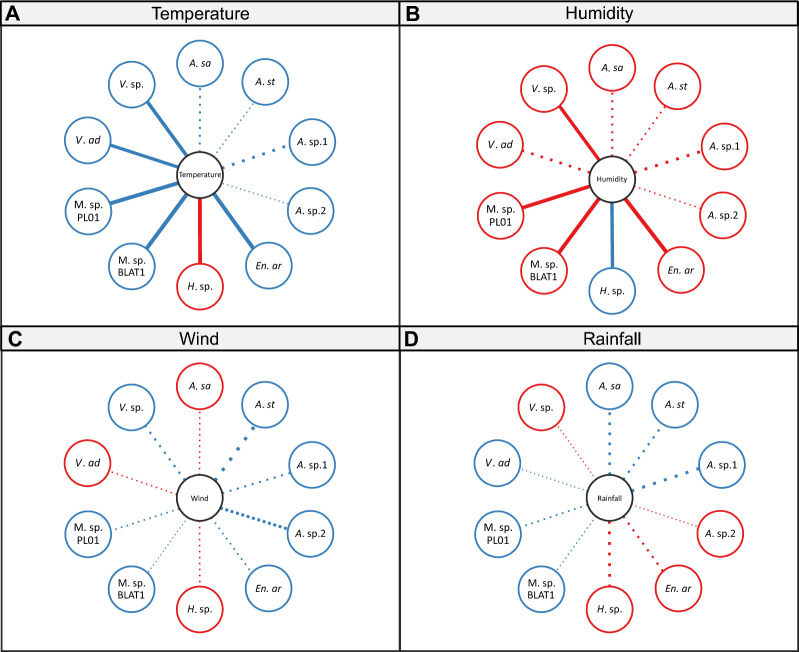
Spearman’s correlation coefficient between the occurrence of microsporidians and environmental factors: **a** temperature, **b** humidity, **c** wind and **d** rainfall. The thickness of line indicates the correlation coefficient. Solid lines indicate statistical significance at *p* < 0.05; dashed lines indicate no statistical significance. For correlation values and their statistical significance, see Additional file 1: Table S6. Abbreviations of microsporidian species: A. sa,* Amblyospora salinaria*; A. st,* A. stimuli*; A. sp.1,* Amblyospora* sp.1; A. sp.2,* Amblyospora* sp.2; En. ar,* Enterocytospora artemiae*; H. sp.,* Hazardia* sp.; M. sp. BLAT1,* Microsporidium* sp. BLAT1; M. sp. PL01,* Microsporidium* sp. PL01; V. ad,* Vairimorpha adaliae*; V. sp.,* Vairimorpha* sp. CHW–2007a

The primary environmental factor affecting the number of microsporidian-positive mosquitoes for each microsporidian species was temperature (Fig. 5; Additional file 1: Table S14). Among the 10 microsporidian species recorded, five (*En. artemiae*, *V. adaliae, Vairimorpha* sp. CHW−2007a, *Microsporidium* sp. BLAT1 and *Microsporidium* sp. PL01) were positively correlated with an increase in temperature (0.656 ≤ ρ ≤ 0.872), and one (*Hazardia* sp.) was statistically significantly correlated with a decrease in temperature (ρ = − 0.821) (Fig. 5a; Additional file 1: Table S14). The remaining microsporidian species belonged only to the genus *Amblyospora*. Although these microsporidians showed a positive correlation with an increase in temperature (0.038 ≤ ρ ≤ 0.508), this result was not statistically significant (Fig. 5a; Additional file 1: Table S14). Owing to the inverse relationship between temperature and humidity, a contrasting relationship was noted for humidity as a determinant; all microsporidia, except *Hazardia* sp., were negatively correlated with humidity (Fig. 5b; Additional file 1: Table S14). Other factors, such as wind and rainfall, had no significant effect on the prevalence of microsporidians in mosquitoes (− 0.152 ≤ ρ ≤ 0.657; − 0.502 ≤ ρ ≤ 0.563) (Figs. 5c, d; Additional file 1: Table S14).

The results of this global (combined for females and males) relationship were in complete agreement with the calculated rho relationship in female mosquitoes, and all correlations and statistical significances were in accordance (Additional file 1: Table S14). The results differed slightly for males, with only three microsporidian species (*En. artemiae*, *Hazardia* sp., *V. adaliae*) noted, and their occurrence was associated with all environmental factors examined in the present study (Additional file 1: Table S14).

### Seasonal potential of microsporidian co-occurrences

**Fig. 6:**
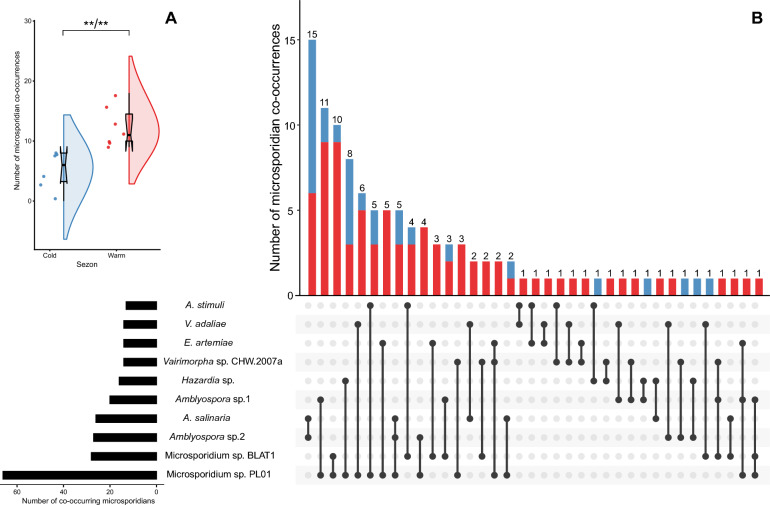
**a** Raincloud plots for the number of microsporidian co-infections during cold and warm months. The dot plots show all the data (jittered horizontally). The box plots show the extremes (whisker tails), interquartile range (box boundaries) and median (horizontal line). The violin plots show the probability density of the data. Symbols (asterisks or 'ns') indicate Bonferroni* p* value (B) and Holm p value (H) (B/H). Double asterisks (**) indicate statistical significance at *p* < 0.01; ns, no statistical significance. **b** UpSet plot of microsporidian co-infections. The bar plot on the left shows the number of co-infected individuals of each microsporidian species. The dot plot on the right shows the number of unique co-infection combinations; connected dots show a microsporidian co-infection combination. The upper bar plot shows the number of infected mosquitoes for each co-infection combination. Bar colour indicates the number of mosquito individuals in which a particular pattern of co-infection was observed; blue indicates cold months, and red indicates warm months

Among the 1023 microsporidian-positive mosquitoes identified, the co-occurrence of at least two different microsporidian species was identified in 109 samples (3.63% of all samples; 10.65% of infected samples). During the warm months, microsporidian co-occurrences were observed in 79 individuals (2.63% compared to all individuals; 7.72% compared to infected individuals). During the cold months, such co-occurrences occurred more than twofold more rarely. We recorded 31 mosquitoes that were positive for > 1 microsporidian species (1.03% compared to all individuals; 3.03% compared to infected individuals). The differences in the number of observed cases within each month were statistically significant within each season (Fig. 6a; Additional file 1: Table S15). A trend toward higher rates of microsporidian co-occurrence during the warm months was noted in both females (*p* < 0.05) and males (*p* < 0.05) (Additional file 1: Table S15).

The co-occurrence of two different microsporidian species was the most common pattern during both the warm and cold months. Three species in one host individual were observed in six females and one male during the warm months, and only in one male and two females during the cold months (Additional file 1: Table S16). *Microsporidium* sp. PL01, the most abundant microsporidian species found in this study, was the primary co-occurring species (63/109 co-occurring individuals, 57.8%). Relatively high numbers of co-occurrences were observed for three *Amblyospora* spp. (*A. salinaria*, *Amblyospora* sp.1 and *Amblyospora* sp.2), and *Microsporidium* sp. BLAT1. Each species was observed with another microsporidian species in a minimum of 20 host individuals, whereas the remaining microsporidian species co-occurred in approximately 12 individuals (range: 13–14) (Fig. 6b; Additional file 1: Table S16).

Overall, 37 combinations of microsporidian co-occurrence were observed (Fig. 6b). Seventeen combinations were present in at least two individuals, eight of which were relatively frequent (in at least 5 individuals) (Fig. 6b; Additional file 1: Table S17). Almost all combinations were noted during warm months (33/37; 89.2%), whereas 15 combinations were observed during colder months. The most common combination in both warm and cold months was the co-occurrence of *A. salinaria* and *Amblyospora* sp.2. Characteristic combinations of co-occurrences during the warm months appeared to involve *Microsporidium* sp. PL01, *Amblyospora* sp.1 (component A = 0.82; *p* = 0.005), *Microsporidium* sp. BLAT1 (component A = 0.9; *p* = 0.005) and *En. artemiae* (component A = 1; *p* = 0.04) (Additional file 1: Table S17). No characteristic combination was observed in mosquitoes during the cold months. Because of the low frequency of microsporidian co-occurrences in females and males divided into seasons, it was not possible to determine the significance of the microsporidian combinations (Additional file 1: Table S15).

## Discussion

### Seasonal dynamics of mosquitoes in western Poland

All mosquito species collected in this study have been reported in Poland previously [95–97]. A significant relationship was noted between average air temperature and dominant mosquito species. During the warmer months (> 19 °C), species belonging to the genus *Ochleratatus*, primarily *O. annulipes* and *O. cantans*, dominated, whereas during the cooler months (< 16 °C), the most frequently occurring species was *Cx. pipiens*. This observation is consistent with previous laboratory [98–100] and field [101] studies, which indicated that, in urban areas, maximum mosquito abundance varies by mosquito species, with the abundance increasing for some mosquito species as air temperature increases. Furthermore, another previous study [101] showed that *Ochlerotatus* spp. mosquitoes were more common at higher temperatures (range: 23.2–25.3 °C than *Cx. pipiens* (approximately 22.1 °C), which is consistent with our results.

Although mosquito abundance and distribution are strongly influenced by anthropogenic factors, such as predation, competition and vector control at the local scale [102], abiotic factors, such as climate and landscape, play a dominant role at larger geographic scales [103]. As a result, increasing global temperatures are shifting mosquito distribution ranges and, thereby, the ranges of pathogens associated with these insects [104–107]. Persistently higher temperatures in Poland will likely affect the longer period of dominance of *Ochlerotatus* and thus increase exposure to pathogenic agents, such as the Eastern equine encephalitis [108], Jamestown Canyon [109], Snowshoe Hare [110], Tahyna [111] and West Nile viruses [108]. *Anopheles messeae* was not collected in our study; but the authors of a previous study did report sparse numbers of *An. messeae* in Poznan [5]. Variations in regional temperatures can have significant implications for the prevalence of malaria worldwide. To date, limited information is available on the presence of *An. messeae* in Poland [5, 111]. However, as the average annual temperature increases, the length of warm months throughout the year increase, creating beneficial conditions for the potential invasion *of An. messeae* in Europe and the Mediterranean regions [112–114].

### Microsporidian occurrence across mosquitoes

It must be noted that the presence of microsporidian DNA does not necessarily result from an infection. It is possible that the detected DNA represents the genetic material of ingested spores, which subsequently move through the digestive tract and never germinate, thus not infecting the host cells [115, 116]. Therefore, in our analyses, we excluded *E. hellem* and *V. ceranae* as infecting factors because they were noted in only < 1% of all analysed individuals (0.1% and 0.57%, respectively) and their ASVs were covered by low numbers of reads. However, there is no empirical basis for the exclusion of *Amblyospora* spp., *En. artemiae*, *Hazardia* spp., *Vairimorpha* spp., *Microsporidium* sp. BLAT1 and *Microsporidium* sp. PL01 because their prevalence was ≥ 1.5% of all analysed individuals. The number of reads for these species ranged from 50 to 51,000, indicating the level of microsporidian infection in the mosquitoes tested, with infection found in at least five mosquito species.

Molecular and histopathological studies have confirmed that at least 150 species of the genus *Amblyospora* infect mosquitoes [3, 5, 117]. Therefore, there is no empirical basis to exclude *Amblyospora* spp. identified in this study from infecting mosquitoes. Similarly, histopathological data shows that *Hazardia* sp. is a mosquito-infecting microsporidium [117, 118]. In our previous study conducted near the city of Poznan, Poland, we observed the presence of *Hazardia* sp. in *Cx. pipiens* females and males as well as in *O. cantans* and *O. sticticus* females [5]. Natural infections of *H. milleri* were observed in *Culex quinquefasiatus* larvae collected in Texas and Louisiana and from *Culex fatigans* collected in Bangkok, Thailand [117, 118]. Therefore, our results confirm that microsporidians belonging to the genus *Hazardia* are associated with mosquito hosts and can be used as indicator species during the cool months.

Crustaceans belonging to order Decapoda and class Branchiopoda, including *Artemia* spp. and *Palaemonetes sinensis*, are the most common hosts of *En. artemiae* [119–122]. In our previous studies using next-generation sequencing (NGS), we detected the presence of *En. artemiae* DNA from various mosquito species [5, 67]. Confirmed infections in various crustaceans have indicated that *En. artemiae* are generalist parasites. Our results suggested that mosquitoes may also be the hosts of *En. artemiae*, but a histological analysis is required to confirm the actual infection. Our data also suggested that *En. artemie* may have low parasite fitness toward mosquitoes. Taken together, *En. artemie* in mosquitoes are moderately virulent and highly infectious parasites with high spore production.

*Microsporidium* sp. BLAT1 has previously been detected only in crustaceans from Lake Baikal, Russia (Qiu et al., GenBank: FJ756034). In our study, we identified this microsporidium in eight mosquito species belonging to the genera *Coquillettidia*, *Culex* and *Ochlerotatus*. Similarly, among the 10 mosquito species representing the same three genera, *Microsporidium* sp. PL01 was detected using molecular methods. The presence of* Microsporidium* sp. BLAT1, and *Microsporidium* sp. PL01 DNA from different mosquito species during different years of mosquito collection strongly suggests that these microsporidians infect mosquitoes.

In the present study, we identified three *Vairimorpha* species: *V. adaliae*, *V. ceranae* *and Vairimorpha* sp. CHW−2007a. We excluded *V. ceranae* from further analysis as it was likely introduced into the mosquitoes through accidental environmental spore inoculation. The other two *Vairimorpha* species were identified using molecular methods in both our previous and current studies, with mosquitoes considered to be hosts for these microsporidia [5, 67]. Thus, *V. adaliae* and *Vairimorpha* sp. CHW−2007a cannot be excluded from infecting mosquitoes and being mosquito pathogens. However, histological and/or quantitative analyses are required to confirm infection.

### Temperature effect on microsporidian occurrence

In the present study, we showed for the first time that temperature has a significant impact on the occurrence of microsporidia in mosquitoes. To date, studies that consider temperature as a factor affecting microsporidians are limited. In one of the first studies in this area, Yan and Larsson [123] reported that the increased prevalence of *Plistophora asperospor* and *Pleistophora crangon* in a natural population of *Holopedium gibberum* in summer was not related to exposure of the parasite to warmer temperatures. These authors stated that the seasonal pattern of parasite prevalence may have been a consequence of host and parasite population dynamics and their interaction, or the result of food stress. Subsequent studies on the effect of temperature on microsporidians included honeybees (*Apis mellifera*) [124, 125], crustaceans (*Artemia franciscana*, *A. parthenogenetica*) [126], flies (*Simulium pertinax*) [127, 128] and mosquitoes [64] as microsporidian hosts. In contrast to the study of Yan and Larson [123], the results of these subsequent studies consistently indicated that the occurrence of microsporidians in their hosts is strongly correlated with temperature.

In the present study, we found that the prevalence of microsporidians in mosquitoes was nearly twofold higher during the warm months than during the cold months. Temperature and humidity appeared to be the primary environmental factors influencing the results. Both of these variables were inversely correlated and equally correlated with the number of microsporidian-positive mosquitoes. We found that as the temperature increased, the number of mosquitoes positive for microsporidians increased and then decreased as the humidity decreased.

Our data, obtained from natural populations, corroborate the results of previous studies, as we observed that mean monthly temperatures of > 19 °C and humidity not exceeding 62% are optimal environmental conditions for microsporidians infecting mosquitoes in Central Europe. However, as the presence of microsporidian DNA is not necessarily due to infection, confirmation of the infection by microscopic analysis is required. In addition, comparing the proliferation efficiency of microsporidians during the warm and cold periods using quantitative analyses would provide further understanding of the infection dynamics. However, notably, the occurrence of *Hazardia* sp. during the cold months was found to be strongly related to its host specificity for *Cx. pipiens*, which was primarily present during the cold months considered in this study.

### Variations in microsporidian presence between males and females

Although our research and data from the literature indicate that temperature is the primary environmental factor affecting microsporidian occurrence rates, variables such as daily fluctuations in temperature, the rate of parasite development and essential elements of mosquito biology, including life stages and sex, should also be considered [45, 47, 105, 129].

Our study showed that the rate of microsporidia occurrence in males was constant at approximately 25% throughout the warm and cold seasons. In contrast, females were more likely to be microsporidian positive during the warm months than during the cold months (50% and 28%, respectively). This observation can be explained by the difference in the lifespans of male and female mosquitoes. In general, females have a longer lifespan than males [130–134], and this difference varies from approximately 1 week for *Cx. quinquefasciatus* [133] and *Cx. fatigans* [130] to 1 month for *Aedes aegypti* [130]. A longer life history can result in a longer exposure to microsporidian spores. In addition, temperature can affect the duration and distance travelled by mosquitoes. The optimum flight temperature has been estimated to range from 15 °C to 32 °C [135]. At lower (10 °C) or higher (35 °C) temperatures, flight is possible but only for short durations. Moreover, higher temperatures affect the frequency of wing beats [136]. In conclusion, the longer lifespan of females and the influence of temperature on their flight activity suggest that these two factors contribute to a higher exposure of female mosquitoes to microsporidian spores during warm months. However, further experiments under controlled conditions are required to confirm this hypothesis.

### Effects of temperature on microsporidians in co-occurrences

The level of co-occurrence of different microsporidian species in the same host in the present study was 3.63%, which is consistent with the level of co-infection (3.6%) noted in our previous study [5]. The results of the present study confirmed a strong co-occurrence relationship between *A. salinaria* and *Amblyospora* sp. 2. In addition, warm months promoted microsporidian co-occurrence: compared with the cold months, we recorded twofold more individuals with at least two microsporidian species during the warm months. Previous research on the co-occurrence of microsporidians has focused on the interactions between microsporidians themselves rather than on the influence of environmental factors that promote co-infection [137–140]. To the best of our knowledge, the present study is the first to show that seasonal climatic factors play an important role in the dynamics of both the occurrence and co-occurrence of microsporidians. Therefore, we cannot refer to the results of other studies. Notably, the increase in the co-occurrence of various species of microsporidians in the same host individual during warm months does not necessarily arise from parasite-host interactions and may result from a greater abundance of spores in the environment. However, we believe that our observations may prompt further studies to address this question.

## Conclusions

In the present study, we demonstrated that climatic factors, such as temperature, determine the seasonal occurrence of microsporidians among mosquitoes. We showed that the prevalence of microsporidians in mosquitoes during warm months was nearly double that during cold months. This observation applies to the dominant species in the population; some microsporidian species have a preference for warm months (*Enterocytospora artemiae*) or alternatively for cold months (*Hazardia* sp.).

Our results suggest that the susceptibility of mosquitoes to parasite occurrence is primarily determined by their activity—and not by the state associated with suboptimal environmental conditions. This observation may be relevant to efforts to biologically control malaria-transmitting mosquito populations, which consider the dual role of microsporidians as natural parasites that directly reduce the vector population and as agents that limit *Plasmodium* development in mosquito tissues.

Although our results are based on DNA data, they are largely consistent with previous findings based on techniques that directly detect parasites, such as spore counting and histochemical techniques, indicating that DNA barcoding is a useful technique that can promptly provide reliable data on microparasites and their hosts.

### Supplementary Information

**Additional file 1: Figure S1:** Correlation plots between the environmental variables: (A) temperature vs. humidity, (B) temperature vs. wind (C), temperature vs. rainfall (D) humidity vs. wind, (E) humidity vs. rainfall, (F) wind vs. rainfall. **Figure S2:** Phylogenetic tree of Microsporidia inferred from BI and ML analyses of concatenated ssu rRNA gene sequence data. Values near branches show Bayesian posterior probabilities (PP) and bootstrap support values (BS) (PP/BS). Black circles: maximally supported; empty circles: supported > 0.95 PP and > 75% BS. Sequences found in this study are in bold. Figure according to Trzebny et al. (2023): 10.1016/j.jip.2022.107873. **Figure S3:** Correlation plots between microsporidian-positive mosquitoes and (A) temperature (°C), (B) humidity (%), (C) wind (m/s), and (D) rainfall (mm). The R value indicates the Pearson’s correlation coefficient statistic, the p value is statistically significant, the shadowed area shows the 95% confidence interval, and the black line is the regression line. The month and year of the data points are indicated according to the legend at the bottom. **Figure S4.** Host mosquito species in which particular microsporidian species were found. Vertical axis: percentage of a given mosquito host.**Table S1:** Daily values of temperature, humidity, wind and precipitation registered in the study area. **Table S2:** Descriptive values (mean, standard deviation, minimum and maximum) of each environmental factor. **Table S3:** Number of mosquitoes collected in each month of the study. **Table S4:** GenBank accession numbers of the COI sequences found in this study. **Table S5:** Percentage of mosquitoes collected in each month of the study. **Table S6:** Correlation values between climatic variables and the occurrence of mosquitoes. **Table S7:** GenBank accession numbers of the V5 18S rRNA gene sequences found in this study. **Table S8:** Number of mosquitoes positive for microsporidian DNA. **Table S9:** Percentage of mosquitoes positive for microsporidian DNA. **Table S10:** Number of mosquitoes divided by sex, positive for microsporidian DNA. **Table S11:** Percentage of mosquitoes divided by sex positive for microsporidian DNA. **Table S12:** Two-way ANOVA F distribution for the relationships between microsporidians detected in mosquitoes and sex of mosquitoes, month (cold-warm) and both factors. **Table S13:** Indicator species analysis for season. Microsporidians that are significant are shown. The “A component” indicates how exclusive the species was for season, where a value of 1 indicates that the species was exclusively found in that group. The “B component” indicates how frequently the given species was found in replicate samples, where 1 means it was found in every sample. **Table S14:** Spearman correlation coefficients (rho) for the relationships between microsporidians detected in mosquitoes and environmental factors. **Table S15:** The number of observed occurrence and co-occurrence in the analysed mosquitoes. **Table S16:** Identified co-occurring microsporidia in each individual. **Table S17:** Frequency of co-occurrence pairs in females and males divided by season. The pairs noted in both warm and cold seasons are shown.

## Data Availability

Sequences generated in this study are available in GenBank under accession nos. ON240798–ON240799, ON240802 ON240817, MN173999, MN174016, MN174018, MT015707, MT015747, MT015750, MT015753, and PP330772–PP330774.
